# Plant Nucleotide Binding Site–Leucine-Rich Repeat (NBS-LRR) Genes: Active Guardians in Host Defense Responses

**DOI:** 10.3390/ijms14047302

**Published:** 2013-04-02

**Authors:** Daniela Marone, Maria A. Russo, Giovanni Laidò, Anna M. De Leonardis, Anna M. Mastrangelo

**Affiliations:** Consiglio per la Ricerca e la Sperimentazione in Agricoltura, Agricultural Research Council-Cereal Research Centre (CRA-CER), SS 16 km 675, 71122 Foggia, Italy; E-Mails: danielamarone@hotmail.com (D.M.); marianna_fg@libero.it (M.A.R.); giovanni.lai79@libero.it (G.L.); annamariadeleonardis@libero.it (A.M.D.L.)

**Keywords:** NBS-LRR genes, gene evolution, plant breeding

## Abstract

The most represented group of resistance genes are those of the nucleotide binding site–leucine-rich repeat (NBS-LRR) class. These genes are very numerous in the plant genome, and they often occur in clusters at specific loci following gene duplication and amplification events. To date, hundreds of resistance genes and relatively few quantitative trait loci for plant resistance to pathogens have been mapped in different species, with some also cloned. When these NBS-LRR genes have been physically or genetically mapped, many cases have shown co-localization between resistance loci and NBS-LRR genes. This has allowed the identification of candidate genes for resistance, and the development of molecular markers linked to R genes. This review is focused on recent genomics studies that have described the abundance, distribution and evolution of NBS-LRR genes in plant genomes. Furthermore, in terms of their expression, NBS-LRR genes are under fine regulation by *cis*- and *trans*-acting elements. Recent findings have provided insights into the roles of alternative splicing, the ubiquitin/proteasome system, and miRNAs and secondary siRNAs in the regulation of NBS-LRR gene expression at the post-transcriptional, post-translational and epigenetic levels. The possibility to use this knowledge for genetic improvement of plant resistance to pathogens is discussed.

## 1. Introduction

Plant responses to pathogenic microorganisms are based on two main mechanisms. The first is the basal defense, based on the actions of the basal immune system, which was first described over 30 years ago [1]. This system can be activated by the so-called elicitors, which are generic signals of the presence of a pathogen, such as bacterial flagellins, lipopolysaccharides or elongation factors, and fungal chitin or heptaglucosides [2]. These are referred to as pathogen-associated molecular patterns (PAMPs), or, more recently, as microbe-associated molecular patterns (MAMPs), because nonpathogenic microorganisms also have PAMPs [3]. The second mechanism is based on the actions of the adaptive immune system, which is composed of resistance (*R*) genes that can specifically recognize host proteins. These are coded by the pathogen *Avr* genes and they confer a resistant phenotype to the plant, as postulated by the gene for gene theory [4]. The *R*-gene products can recognize the products of the *Avr* genes directly or indirectly. For the latter, the guard and decoy hypothesis propose that the *Avr* gene products result in “perturbation” of the components of the adaptive immune system, separate from the R proteins. It could be an accessory protein, which may be its virulence target (guard model) or a structural mimic of such a target (decoy model) [5]. This perturbation acts as a trigger for the activation of these *R* genes. An example is the *Pseudomonas syringae Avr* gene *AvrPphB*, which codes for a protease that can in turn cleave a host protein kinase. This cleavage is detected by the cognate R protein (resistance to *Pseudomonas syringae* 5; RPS5), which then becomes active [6]. While the MAMP receptors are relatively stable and heritable, the components of the adaptive immune system are subject to diversification and selection in somatic cells of individuals, so that there is continuous co-evolution of plants and pathogens [3].

At least five different classes of *R* genes are known to date [7]. The most numerous *R*-gene class is represented by the members of the gene family that code for proteins containing a nucleotide-binding site (NBS) and leucine-reach repeats (LRRs) [8]. The NBS domains are involved in signaling, and they include several highly conserved and strictly ordered motifs, such as the P-loop, kinase-2 and Gly-Leu-Pro-Leu motifs [9]. LRRs are highly adaptable structural domains that are devoted to protein-protein interactions, and these can evolve very different binding specificities. LRRs are under diversifying selection [10,11], especially at the level of the predicted solvent-exposed residues. In this region, these NBS-LRR proteins not only lack conservation, but they are significantly more diverse than expected from random genetic drift. This suggests that there are selective pressures that promote the evolving of new pathogen-specificities, for the recognition of different pathogen Avr proteins. Clustering of NBS-LRR genes due to tandem duplications and ectopic duplications followed by local rearrangements and gene conversion are also important processes that influence the evolution of this gene family.

The presence of different domains at the *N*-terminal portion of the NBS-LRR proteins classifies these NBS-LRR gene products into two subgroups: the TIR-NBS-LRR (TNL) proteins that contain a Toll-like domain, and the CC-NBS-LRR (CNL) proteins that are characterized by a coiled-coil domain; this subdivision is, however, not always precise. With several hundreds of members, the NBS-LRR genes are one of the most numerous gene families in plants, as has also been demonstrated by recent studies carried out with species with a sequenced genome. At the same time, there are huge differences across species in terms of the numbers and organization of their subgroups.

This review focuses on the extent and role of the NBS-LRR resistance-gene family in plant responses to pathogens in this era of “-omic” technologies. Particular attention is paid to the following aspects: (i) genomic organization of the gene family in plant species in which the genome has been sequenced; (ii) molecular aspects of the actions of NBS-LRR genes in plant responses to pathogens, and the mechanisms of the regulation of their expression, from transcriptional to the post-translational level; (iii) coincidence of NBS-LRR genes with *R* loci, and the implications of this knowledge of the NBS-LRR gene family for the genetic improvement of crops in terms of their resistance to pathogens, without increasing the metabolic cost of resistance.

## 2. Genomic Organization and Evolution of NBS-LRR Genes in the Plant Genome

### 2.1. Genomic Organization and Evolution of NBS-LRR Genes

Comparative genomic analyses have indicated that plant genomes can encode several hundreds of NBS-LRR genes, and that there is a great diversity in the number and distribution of the subclasses of these genes. To date, a large number of NBS-encoding sequences have been isolated from various plant species through genome-wide analyses: from about 50 in papaya and *Cucumis sativus*[12,13], to 653 in *Oryza sativa*[14]. More details are shown in Table 1, that reports the number of NBS-encoding genes identified in different species up to now. These data are subject to a continuous evolution, as new genomic sequences are produced very rapidly, but they provide an idea of the size and organization of this gene family in plant genomes.

Except for papaya, in which NBS-LRR genes are well distributed across the linkage groups [12], the chromosomal distribution of NBS-LRR genes appears to be very irregular in most species studied, with some chromosomes characterized by many more NBS-LRR genes than others. With potato, for example, the greatest numbers of NBS-LRR genes are found on chromosomes 4 and 11 (about 15% of the mapped genes), with the smallest number on chromosome 3 (1%) [15,16]. Also, in *Brachypodium distachyon*, chromosome 4 contains about one-third of the total NBS-LRR genes that have been identified [9]. Conversely, chromosome 4 is less represented in *Brassica rapa*, where chromosomes 3 and 9 contain more than half of the mapped NBS-LRR genes [17], and in *Lotus japonicus*, where the more represented chromosomes for the mapped NBS-LRR genes are chromosomes 2 and 3. Kang *et al.*[18] reported that in the soybean genome, chromosome 16 has the highest number of NBS-LRR genes. Finally, in *Medicago truncatula*, more than 54% of NBS-LRR genes are encoded by chromosomes 3, 4 and 6 [19].

As indicated above, NBS-LRR genes are usually divided into two main subclasses, as CNL and TNL genes, and the distribution of NBS-LRR genes into these two subgroups is not comparable across different plant species. The most striking example is the near-total absence of TNL genes in monocotyledons. TNL genes are instead present in the dicotyledon genome, and often in greater numbers compared to CNL genes. The genomes of species like *Arabidopsis thaliana*, *Arabidopsis lyrata* and soybean contain from two-fold to six-fold more TNL than CNL genes [21,24], with the opposite seen for potato [15,16] and *Medicago truncatula*[19], with their larger numbers of CNL genes.

Within the genome, NBS-LRR genes are organized either as isolated genes or as linked clusters of varying sizes that are thought to facilitate rapid *R*-gene evolution [25]. These clusters can be divided into two types based on their phylogenetic relationships: (i) clusters that contain NBS-LRR genes that have undergone tandem duplication, whereby NBS-LRR genes that derive from these events are seen to group together in species-wide gene trees; and (ii) mixed clusters that contain NBS-LRR genes from different branches of species-wide trees [26], where the genes belonging to the same cluster were derived from ectopic duplication, transposition, or large-scale segmental duplication, with subsequent local rearrangements [27]. This latter clustering phenomenon involves a number of NBS-LRR genes, and although it varies depending on the species, it often involves the majority of the NBS-LRR gene family members. As examples: Li *et al.*[18] reported that in the entire *L. japonicus* genome, 38.2% of the mapped NBS genes are located as eight clusters, with uneven chromosomal distribution; in the rice genome, 50% of the identified NBS-LRR genes are clustered [28] and in *B. distachyon*, 51% are clustered [9]; and higher rates are found in other species, like potato, where 73% of the mapped NBS-LRR genes are grouped into 63 clusters [15], and like *M. truncatula*, where nearly 80% are clustered [19]. At the same time, the proportion of gene clustering can also be different in related species. In a recent study, Guo *et al.*[21] identified 159 and 185 NBS-LRR genes in *A. thaliana* and *A. lyrata*, respectively, with the levels of genes organized into clusters for these two species of 71.1% and 63.8%, respectively. Also, even though *A. thaliana* has a lower number of NBS-LRR genes per cluster, these are grouped in a higher number of clusters with respect to *A. lyrata* (38 *vs.* 35). In potato, TNL genes are widely distributed throughout the entire genome, except on chromosomes 3 and 10 [15], and they are more widely dispersed than CNL genes, which are more frequently found in clusters. An explanation for this relates to the predominance of CNL genes in the potato genome (85%), with respect to TNL genes. In some cases TNLs are located near CNL clusters, which can in turn be grouped into larger super clusters [16].

Although the majority of NBS-LRR clusters are composed of similar sequences, many clusters also contain some phylogenetically distant NBS genes. Indeed, about 25% of *M. truncatula* clusters include both TNL and CNL genes. The presence of heterogeneous NBS-LRR clusters is similar also in rice and *Arabidopsis*, where about 25% of NBS-LRR clusters are phylogenetically mixed [19]. A similar rate of mixed NBS-LRR clusters is seen for potato [15].

### 2.2. Pseudogenes

Pseudogenes are commonly defined as sequences that resemble known genes but that cannot produce functional proteins. Pseudogenes can originate through the same mechanisms as protein-coding genes, followed by the subsequent accumulation of disabling mutations (e.g., insertions, deletions, and/or substitutions) that disrupt the reading frame or lead to the insertion of a premature stop codon [29].

A variable number of *R* pseudogenes have been identified in different plant species; these are highly similar to NBS-LRR genes at sequence level, although their sequences are partial or they lead to the production of partial proteins. Low levels of pseudogenes have been shown for *A. thaliana* (8.05%) [20] and *M. truncatula* (14.7%) [19]. A higher level was found in polyploid cotton (24.6%) [30]. However, a role for the level of ploidy in accumulation of the pseudogenes in the genome cannot be suggested, as other studies have reported even higher levels of NBS-LRR pseudogenes in diploid species. A clear example is seen with the rice genome, in which 47.6% and 55.7% pseudogenes have been identified in the Nipponbare and 93–11 genotypes, respectively [31]. This study showed that the number of total predicted NBS-LRR genes is much higher (nearly 700 genes) than that reported for the same cultivars by Yang *et al.*[28] (nearly 500), mainly because partial genes were also considered by Luo *et al.*[31]. In most cases, pseudogenes are characterized by large deletions [31]; these can be produced by transposition events and exon skipping, with frameshift mutations also explaining the formation of truncated mRNAs and proteins. This was shown for the potato genome, in which a level of 41.6% pseudogenes was reported recently [16]. In some cases, pseudogenes differ from functional NBS-LRR genes in terms of the length of the NBS domain, which can be excessively shortened, as shown in *L. japonicus*[18] and *B. distachyon*[9]; here, the NBS motifs are too short or too divergent with respect to the functional NBS.

The number of pseudogenes also varies according to the NBS-LRR subgroup (CNL, TNL), and this difference can reflect the relative abundance of CNL and TNL genes. As an example, out of 179 pseudogenes identified in potato, 156 (87%) belong to the CNL group and 23 (13%) to the TNL group, but this was expected as 85% of the total NBS-LRR genes are CNL and only 15% are TNL [16].

For the genome distribution of pseudogenes, truncated NBS-LRR genes have often been seen to be located adjacent to complete NBS-LRR genes, within 100 kb, and therefore distributed in the same way as functional NBS-LRR genes; indeed, these pseudogenes are also clustered on specific chromosomes, as seen for *M. truncatula*[19] and potato [16].

Pseudogenes are usually considered as nonfunctional genes that will be eliminated from the genome, or reservoirs of genetic diversity that can be accessed through recombination or gene conversion [32]. There is evidence of expression of pseudogenes in some species, including for rice [33], pine [34] and *M. truncatula*[19]. In particular, some pseudogenes identified in these species have near-perfect (99%–100% identity) matches in EST databases. Furthermore, functions can also be ascribed to partial NBS-LRR proteins. In mouse, an expressed pseudogene has been confirmed to be involved in the stability of the mRNA of its functional homolog through the local silencing system [35]. Lozano *et al.*[16] proposed a role as adaptor molecules for pseudogenes identified in potato, whereby they can act as recruiters of or interact with other NBS-LRR proteins. The same hypothesis was formulated for the truncated *R* genes identified in *Populus*[22]. Truncated R polypeptides can originate also by alternative splicing, and a role in promoting disease resistance has been demonstrated for many of these, as reviewed by Mastrangelo *et al.*[36].

### 2.3. Evolution of NBS-LRR Genes in Plants

NBS-LRR genes represent one of the most numerous and ancient gene families in plants. Mechanisms like duplication, unequal crossing over, ectopic recombination, gene conversion, and diversifying selection have been proposed to have contributed to the structures of *R* gene clusters and to the evolution of resistance specificities [37–39]. However, the rate of evolution and the effects of selection are not homogeneous at the level of the different domains within each gene sequence, and neither for the members of the different subgroups within the gene family. Regarding this first aspect, the gene conversion rate appears to have been higher for LRR domains than for NBS regions, which appear to be subject to purifying selection [40]. The high variability of LRR domains is related to their role in the recognition of specific *Avr* gene products, to promote plant resistance to pathogens. Indeed, elevated ratios of nonsynonymous to synonymous nucleotide substitutions have been found in LRR domains; this indicates that diversifying selection acts on LRR domains to maintain variations in the solvent-exposed residues [41]. To have an idea of the variations at the level of LRR domains, there are, on average, 14 LRRs per protein, and often five to 10 sequence variants for each repeat; therefore, in a species like *Arabidopsis*, there is the potential for well over 9 × 10^11^ variants [42].

For the variability across NBS-LRR genes, it is often possible to recognize genes with two patterns of evolution [40]: type I genes have evolved rapidly, with frequent gene conversions between them, whereas type II genes have evolved slowly, with rare gene conversion events between clades. This heterogeneous rate of evolution is consistent with a birth-and-death model of *R* gene evolution, in which gene duplication and unequal crossing over can be followed by density-dependent purifying selection [42]. One of the factors that influences this gene conversion is the clustering of *R* genes, as larger clusters provide more potential donor sequences. However, this is not always true, as it can depend on the mating system. Guo *et al.*[21] showed significant positive correlations between cluster size and gene conversion frequency in *A. thaliana* but not in *A. lyrata*, where the median length of conversion tracts is higher, probably due to the lower rate of recombination of out-crossers with respect to related selfers.

An interesting question arises when NBS regions and LRR domains become fused into the same protein. Only independent NBS and LRR domains have been found in the genomes of bacteria, archaea, protists and algae [43]. However, in a recent study, Xue *et al.*[44] reported NBS-LRR genes in two bryophyte species: the moss *Physcomitrella patens* and the liverwort *Marchantia polymorpha*. As well as the classical CNL and TNL subgroups, they defined two novel classes of NBS-encoding genes. The first was identified in the *P. patens* genome, and it showed a protein kinase domain at the *N*-terminus (PK-NBS-LRR; PNL). The second was identified in the *M. polymorpha* genome, and it was characterized by an a/b-hydrolase domain at the *N*-terminus (hydrolase-NBS-LRR; HNL). In light of these results, NBS-LRR genes appear to have originated and been selected to allow early lineages of land plants to cope with specific pathogens. The CNL class was shown to be divergent from TNL, PNL and HNL in bryophytes, as the liverwort *M. polymorpha* CNL genes and some of the moss *P. patens* CNL genes form a strongly supported monophyletic group [44]. Furthermore, CNL from monocotyledons and dicotyledons tend to cluster together, which suggests that the CNL group originated before the divergence of the monocotyledons and dicotyledons [23,42]; in contrast, TIR domains are absent in cereal genomes, and they are most likely to have been lost from the cereal species, rather than to have arisen later, after the monocotyledon/dicotyledon separation [45].

The number of single-copy and duplicated NBS-LRR genes reflects the small or large scale duplication events that have occurred during genome evolution. In *Arabidopsis*, Meyers *et al.*[20] reported that local and distant duplications of TNL and CNL genes are responsible for the separation of two distinct clusters, which is compatible with the duplication of the entire genome early in the evolution of *Arabidopsis*. Similarly, large duplication blocks have also been identified in *M. truncatula*, the genome of which underwent large-scale genomic duplication [19]. A clear separation between two groups has been identified also in *Brassica rapa*, despite the whole-genome-triplication event that occurred 11 to 12 million years ago, after speciation [17]. Furthermore, the number of NBS-LRR genes is slightly greater than in *Arabidopsis*, in which, instead, duplication of the genome occurred. Probably, roughly half of the NBS-LRR genes generated after the triplication of the *B. rapa* genome have now been lost, demonstrating this rapid birth-and-death system in the *Brassica R*-gene family. A similar loss of *R* genes has probably occurred for the *Populus* genome, for which segmental duplications and subsequent chromosomal rearrangements have only resulted in 5% amplification of NBS-LRR genes [22].

Often, the duplication of genomic regions that contain NBS-LRR genes corresponds also to functional redundance. The presence of genes with redundant functions within duplicated regions has been demonstrated in the soybean genome [24]. This study reported that 91 NBS-LRR genes were within 10 duplicated genomic regions, and that these regions contained duplicated disease resistance quantitative trait loci (QTL). Moreover, in some cases, there was a similar copy number of NBS-LRR genes on each side of the duplication, whereas there were distinct numbers of genes in other regions for both of the duplicated sides. This will probably have been due to tandem duplication that occurred independently on one side of a duplicated region.

In conclusion, the uneven and clustered distributions of NBS-LRR–encoding genes and the discovery of duplicated redundant genes in recent plant genome-wide studies have contributed to provide new insight into the generation of novel resistance specificities and to the expansion of this gene family through gene-duplication events (tandem or segmental duplications), transpositions, chromosomal rearrangements (e.g., ectopic translocations), unequal crossing over, and diversifying selection.

## 3. Regulation and Function of NBS-LRR Genes

### 3.1. Molecular Basis of Resistance Responses Induced by NBS-LRR Proteins

The molecular basis of the responses to pathogens in plants depends on the kind of pathogen. In general, R–Avr interactions lead to the hypersensitive responses, which are aimed at the restriction of pathogen growth at the primary infection site and at programmed cell death. This kind of reaction needs to be tightly regulated to avoid tissue damage. Another response, termed systemic acquired resistance, is triggered following activation of hypersensitive responses. Systemic acquired resistance results in broad-spectrum and systemic resistance, and it is characterized by increased salicylic acid accumulation and increased expression of pathogenesis-related genes [46,47]. In the case of virus infections, NBS-LRR genes can drive phenotypic responses that are divided into hypersensitive-response resistance and extreme resistance that shows strong resistance against virus infection [48]. Virus multiplication is restricted to a single-cell level in extreme resistance, in which necrotic local lesions do not develop at the primary infection site. *Rx* gene (CNL)-mediated resistance to potato virus X in potato shows features of extreme resistance [49], while *N*-gene-mediated resistance to tobacco mosaic virus in tobacco and *HRT* (hypersensitive response to turnip crinkle virus)-gene-mediated resistance to turnip crinkle virus in *A. thaliana* are examples of hypersensitive-response resistance. The type of resistance can also be linked to the expression level of the *R* gene; indeed, overexpression in *A. thaliana* of *RCY1* (resistance to cereal mosaic virus (Y)), an allelic form of *HRT*, confers an extreme-resistance phenotype [49].

Of note, despite the specificities of the interactions between the Avr and R proteins, there are no clear specificities in the reactions of NBS-LRR proteins to particular pathogen types. An example is that allelic forms at the same NBS-LRR locus can confer resistance to different strains, and even to different classes of pathogens. The Arabidopsis proteins RPP8 (recognition of peronospora parasitica 1), HRT and RCY1 are encoded by different alleles of the same locus, and these confer resistance to an oomycete and two different viruses, respectively [50–52]. Similarly, the potato Rx and Gpa2 (*Globodera pallida* 2) proteins are highly similar, but these recognize a virus and a nematode, respectively [53,54].

As mentioned above, the specificity of effector recognition is due to the LRR domains, which have been shown to be under diversifying selection to ensure co-evolution with pathogen effectors [55]. As well as the well-known NBS and LRR domains, some small conserved regions have also been identified that form the “nucleotide-binding adaptor shared by APAF-1, R proteins, and CED-4” (NB-ARC) domain, which consists of the NBS and two ARC domains (ARC1, ARC2) [3]. Many highly conserved motifs have been identified in the ARC domain, and these have been studied in detail from a functional point of view. The P-loop motif in NBS is required for nucleotide binding, and mutations to this motif lead to loss-of-function of several NBS-LRR proteins [56]. Auto-activation of many NBS-LRR proteins is instead determined by mutations in the MHD (methionine–histidine–aspartate) motif, which is located in the ARC2 domain and is involved in nucleotide-dependent conformational changes [57].

The current knowledge of the molecular mechanisms of action of NBS-LRR proteins suggests a functional model in which the LRR domain controls the molecular state of the NB-ARC domain [58]. Interactions among domains of the potato CNL protein Rx have been demonstrated, as well as modifications of these interactions, following stimulation by a pathogen Avr protein. Therefore, a model has been proposed that is sometimes referred to as the “jackknife” model, in which the pathogen effector causes disruption of the intramolecular associations, which frees the CC, NB and/or LRR domains for them to take up a “recognition-competent conformation” in which the different domains can interact with other proteins [59]. Further evidence has shown that the presence of a bound nucleotide is required for an NBS-LRR protein to reach this state [60].

Interestingly, the swapping of LRR domains between closely related paralogs often results in constitutive activation of NBS-LRR proteins [61–63]. Therefore, the NBS and LRR domains must have co-evolved to maintain the inhibition of NBS-LRR protein auto-activation. A detailed functional analysis was carried out very recently for the *Arabidopsis* CNL RPS5 disease-resistance protein. RPS5 is activated by AvrPphB-mediated cleavage of the protein kinase PBS1. Qi *et al.*[55] showed that substitution of the CC domain of RPS2 for the CC domain of RPS5 did not alter the RPS5 specificity, and only moderately reduced its ability to activate programmed cell death, which suggests that the CC domain does not have a direct role in the recognition of PBS1 cleavage. *C*-terminal truncations of RPS5 have revealed that the RPS5 LRR domain functions to suppress RPS5 activation in the absence of PBS1 cleavage, and promotes RPS5 activation in its presence.

Nucleotide-dependent conformational changes might, in turn, induce oligomerization, or they might instead provide a scaffold for activation of downstream signaling components. Oligomerization that is dependent on a functional NBS has been shown for the tobacco N protein [64]. For some CNL proteins, the CC domain alone has been shown to sometimes be able to trigger cell death. Some examples are: the Arabidopsis *RPS2*, *RPS5*, *RPM1* (resistance to *P. syringae* Pv *Maculicola* 1) and *ADR1* (activated disease resistance 1); *Nicotiana benthamiana NRG1* (N requirement gene 1); and barley *MLA10* (mildew*-*resistance locus A 10) genes [6,65–68]. Otherwise, for Rx, which is a typical CCEDVID-NB-LRR subtype R protein, its central NBS, and not its N-terminal CCEDVID domain, is sufficient to induce cell death [69].

An important factor for the function of NBS-LRR proteins in the promotion of resistance to pathogens is their subcellular localization. Several R proteins have been shown to localize to both the cytoplasm and the nucleus, even if no clear nuclear localization signals can be identified in most of the R protein sequences. Indeed, in many cases, the R proteins accumulate in the nucleus in response to pathogen infection [70,71], and this nuclear localization has been shown to be essential for the resistant phenotype determined by the barley MLA10, tobacco N, and *Arabidopsis* RPS4, RRS1-R (resistance to *Ralstonia solanacearum*-R) and SNC1 (suppressor of npr1-1, constitutive 1) proteins [70,72–75]. Recent evidence has indicated that uncoupling of the immune response from cell-death signaling is linked to the nucleo-cytoplasmic localization of R proteins. In a recent study, Bai *et al.*[76] showed that barley MLA10 activity in cell-death signaling is suppressed in the nucleus but enhanced in the cytoplasm, while the MLA10 in the nucleus is sufficient to mediate disease resistance against powdery mildew fungus. Furthermore, enforced retention in the cytoplasm has reinforced the role of cytoplasmic MLA10 in cell-death signaling. These data suggest a bifurcation of MLA10-triggered cell death and disease resistance signaling in a compartment-dependent manner. The role of MLA10 in the nucleus was found to be associated to specific intramolecular and intermolecular interactions. A recent study has also revealed that the CC of MLA can form a rod-shaped homodimer in solution, and that MLA dimers define the minimal functional unit that is required for triggering cell death in barley and *N. benthamiana*[68]. Furthermore, in the nucleus, MLA10 interacts with WRKY transcription factors that act as repressors of MAMP-triggered basal defenses. This interaction is therefore believed to remove this WRKY-mediated check on MAMP-induced defenses, which are thereby expressed more strongly and rapidly, leading to highly effective defense and hypersensitive responses.

**Figure 1:**
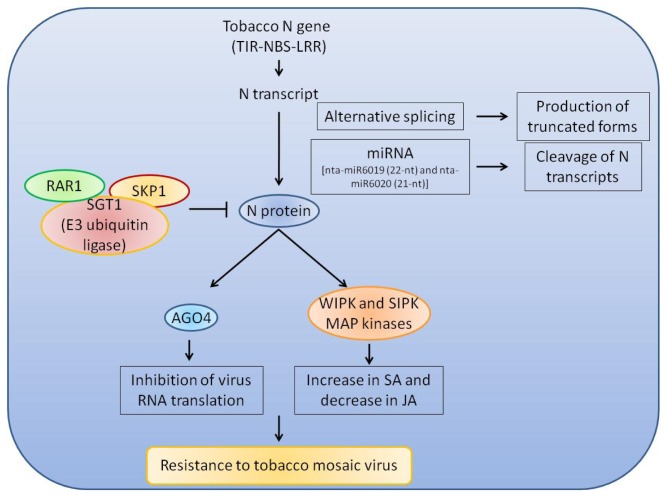
A summary of the mode of action and mechanisms regulating the *N* gene expression at different levels. SA: salycilic acid; JA: jasmonic acid.

Some information is also available on the mechanisms that control NBS-LRR-mediated resistance to viruses. An antiviral response that inhibits the translation of virus-encoded proteins in *N. benthamiana* has been shown for the N protein [77]. In particular, upon activation of the NBS-LRR protein, viral transcripts can accumulate but do not associate with ribosomes, and the inhibition of this interaction is mediated by Argonaute4-like genes. Argonaute proteins have been implicated in small (s)RNA-mediated RNA degradation, and in degradation-independent translational control, and therefore their engagement in the specific translational control of viral transcripts has been proposed as a key factor in virus resistance mediated by NBS-LRR proteins. The action of the *N* gene also involves a mitogen-activated protein kinase cascade. Recent evidence has showed that the mitogen-activated protein kinases WIPK and SIPK function to negatively regulate the local resistance to tobacco mosaic virus accumulation, although they positively regulate systemic resistance. The use of tobacco cultivars that lack or have the *N* gene has suggested that the function of WIPK and SIPK in resistance responses of tobacco plants to tobacco mosaic virus is regulated by the *N* gene [78]. A summary of mechanisms regulating the *N* gene is presented in Figure 1.

### 3.2. Regulation of Expression and Activity of NBS-LRR Genes

NBS-LRR genes need to be finely regulated to ensure correct resistance responses, while limiting their metabolic cost and any detrimental effects on plant growth. This regulation is a very complex process, and it takes place at different levels, from the synthesis/processing of transcripts, to the regulation of protein stability.

Regulation at the transcriptional level is aimed at regulation not only of the quantity of mRNA, but also the quality, as in some cases, different transcript forms can be produced from the same gene by alternative splicing. Based on literature data, several *R* genes are regulated by such alternative splicing of their transcripts. Many cases of regulation of plant-disease resistance genes have been associated to TNLs, with some examples reported for CNLs in cereals [36]. The regulation by alternative splicing of *R* genes mainly acts through the induction of the synthesis of protein forms that are characterized by different combinations of functional domains. Many *R* gene transcripts have been shown to be produced in truncated forms that contain only one or two of the domains present in the full-length transcripts. This is the case for the Arabidopsis *RPS6* (resistance to *P. syringae* 6) gene, for which three alternative transcripts can be produced. While the full-length protein contains three functional domains (TIR, NBS, LRR), the two alternative transcripts are characterized by a premature termination codon and they encode truncated proteins in which there are only one or two domains [79]. Other examples were reviewed in detail by Mastrangelo *et al.*[36]. Interestingly, for many truncated R proteins produced via alternative splicing, positive effects on resistance have been demonstrated, as for the *RLM3* (resistance to *Leptosphaeria maculans* 3) [80], *N*[81], *RPP1*[82], and *RPS5*[8] genes, and for the *RPS4* gene, an *Arabidopsis* TNL gene that is involved in resistance to bacterial pathogens that express the Avr Rps4 in a specific manner [83].

Thus, positive roles in resistance for truncated proteins can be obtained by the alleviation of self-inhibition of the full-length proteins [84] or by their functioning as adaptors for downstream signaling events [85]. How this kind of regulation is carried out is still not clear. In a recent study, Xu *et al.*[86] analyzed the function of the Arabidopsis *MOS14* (modifier of sncl-1,14) gene that codes for the nuclear import receptor for serine-arginine-rich proteins, which are proteins that are involved in different aspects of RNA metabolism. Loss of function of *MOS14* results in altered splicing patterns of *SNC1* and *RPS4* and compromised resistance mediated by snc1 and RPS4, which suggests that the nuclear import of serine-arginine-rich proteins by MOS14 is required for the correct splicing of these two *R* genes, and is important for their functions in plant immunity. The production of diverse protein products can facilitate the genetic evolution of resistance to newly evolved pathogen races that express new effector molecules that can overcome plant resistance. In light of this, together with gene duplication, alternative splicing can participate in the amplification of resistance-gene variation and complexity, which will help plants to cope with biotic stress in plant–pathogen co-evolution [36].

In plants and other organisms, small RNA (sRNA) systems mediate gene silencing and can affect genome integrity, gene regulation, and antiviral defense [87]. MicroRNAs (miRNAs) are versatile regulators of gene expression in plants and animals. MiRNAs are 21 to 24 nucleotides long and are processed by the Dicer nuclease from long RNA precursors with base-paired fold-back structures [88]. The single-stranded forms of miRNAs form ribonucleoprotein complexes with Argonaute (AGO), which can bind by base pairing to a target RNA [89,90]. MiRNAs mediate gene silencing by acting as a negative switch, or by promoting tighter regulation of the expression of a gene. Interestingly, miRNAs and their precursor can move through the plasmodesmata and regulate biological responses in adjacent cells or in separate roots and shoots [91,92]. sRNA-mediated transcriptional gene silencing and posttranscriptional gene silencing have been implicated in the regulation of host defenses against pathogens [93]. Li *et al.*[94] identified miRNA progenitor gene precursor transcripts, and two miRNAs [nta-miR6019 (22-nt) and nta-miR6020 (21-nt)] that guide the cleavage of transcripts of the immune receptor N. *N*-mediated resistance to tobacco mosaic virus is attenuated when N is co-expressed with nta-miR6019 and nta-miR6020. Furthermore, using a bioinformatics approach, six additional 22-nt miRNA and two 21-nt miRNA families from three *Solanaceae* species were identified; these can cleave transcripts of predicted functional *R* genes and trigger the production of phased secondary 21-nt small interfering (si)RNAs. In a study carried out with tomato, Shivaprasad *et al.*[95] analyzed datasets of sRNAs and identified a regulatory cascade that can affect disease resistance. The initiators of the cascade belong to an unusually diverse superfamily, and they target the coding sequence for the P-loop motif in the mRNA sequences for disease-resistance proteins with NBS and LRR motifs. In particular, miR482 targets mRNAs for NBS-LRR disease-resistance proteins with CC domains at their N terminus. Following this interaction, the mRNA decayed and there was production of secondary siRNAs. At least one of these secondary siRNAs targets other mRNAs of a defense-related protein [95]. Altogether, these results demonstrate a conserved role for miRNAs and secondary siRNAs in NBS-LRR immune receptor gene regulation and pathogen resistance in *Solanaceae*.

Plant responses to pathogens need to be blocked in the absence of the pathogen, to avoid auto-immunity, which can be detrimental to plant growth and development. Arabidopsis *SNC1* encodes a TNL-type R protein [84]. The snc1 mutant, which is characterized by a gain-of-function mutation that is located in the region between the NBS and LRR domains, shows constitutively activated downstream defense responses, such as the accumulation of high levels of salicylic acid, and constitutive expression of pathogenesis-related genes [96], a phenotype that is associated with a dwarf morphology. The function of some proteins that have been studied consists of blocking the action of R proteins in the absence of the pathogen. Some of these proteins have been shown to interact with molecular chaperons. In Arabidopsis, the RAR1 (required for Ml-a12 conditioned resistance), SGT1 (suppressor of G2 allele of skp1) and HSP90 (heat-shock protein 90) proteins are involved in the correct folding of NBS-LRR proteins. Accumulation of the barley MLA, potato Rx, and Arabidopsis RPM1 and RPS5 proteins was reduced when RAR1 function was compromised [97–99]. Compromising the activity of HSP90 also resulted in reduced accumulation of several R proteins, including RPM1, RPS5 and Rx [99–101]. In a recent study, Li *et al.*[102] identified an evolutionarily conserved protein, SRFR1 (suppressor of rps4-RLD 1), that interacts with SGT1 and has a role in repression of the immune responses; these are indeed constitutively activated in loss-of-function mutations of SRFR1.

SGT1 is an E3 ubiquitin ligase, which is a component of the ubiquitination system, a post-translational regulation pathway in which ubiquitin is bond to lysine residues of target proteins and promotes their degradation by the 26S/proteasome. It has been shown by mutational analysis that AtSGT1b is required for *A. thaliana* resistance against *Peronospora parasitica*[103,104]. As interactions between SGT1 and RAR1 have been demonstrated in *Arabidopsis*, a model has been proposed in which SGT1 participates in the degradation of RPM1 in order to control the hypersensitive-response lesion size and response amplitude at a site of infection [105]. The involvement of RAR1 and SGT1 in defense mechanisms has also been highlighted in several other plants [105]. Another example of E3 ubiquitin ligase being involved in resistance to pathogens is seen for CPR30 (constitutive expresser of pathogenesis-related genes 30), which was isolated in Arabidopsis. Mutations in the *CPR30* gene resulted in a dwarf morphology, constitutive resistance to the bacterial pathogen *P. syringae*, and dramatic induction of defense-response gene expression. The growth defect of cpr30 was suppressed fully by eds1-1 (enhanced disease susceptibility 1) and ndr1-1 (nonrace-specific disease resistance 1-1) [106]. As EDS1 and NDR1-1 constitute a regulatory hub that is essential for basal resistance to invasive biotrophic and hemi-biotrophic pathogens, and as they are also recruited by TNL and CNL proteins, respectively, to signal isolate-specific pathogen recognition, CPR30 might be involved in the regulation of both the TNL-type and CNL-type *R*-gene-mediated defenses [46].

## 4. NBS-LRR Genes: A Resource for Plant Breeding

### 4.1. Co-Localization of NBS-LRR Genes with *R* Loci

The map-based cloning of *R* genes has demonstrated in many cases the role of NBS-LRR genes in the promotion of resistance against a number of pathogens. In some cases, the molecular mechanisms of this action have been defined (see Section 3.1.). However, the experiments needed here are very time consuming and laborious, especially for species that are characterized by a complex genome. Alternatively, a possible role in disease resistance can be proposed for a much higher number of genes belonging to this family based on their co-segregation with genetically mapped *R* loci.

These kinds of analysis can be carried out by the identification of NBS-LRR genes in sequenced genomes, and then with comparisons of their positions with those of the *R* loci. At the same time, it is possible to investigate this correspondence even for species where the genome has not been sequenced, through genetic maps that are rich in molecular markers that are designed on the basis of NBS-LRR genes. An example of the first kind of approach is represented by a study that was carried out in soybean. Kang *et al.*[24] analyzed NBS-LRR genes that co-localized with disease-resistance QTL, and they observed that about 63% of the disease-resistance QTL were located in the 2-Mb regions that flank the NBS-LRR genes. In particular, a region on chromosome 6 contained many clustered NBS-LRRs and was associated to seven resistance QTL, four of which conferred fungal resistance, and three of which conferred nematode resistance. Another example is chromosome 16, where 40 NBS-LRR genes were mapped. In this case, 19 resistance QTL fell within the 2-Mb regions flanking them, as 14 QTL for fungal resistance, and five QTL for nematode resistance. A similar approach led by Shang *et al.*[14] identified relationships between NBS-LRR pseudogenes and blast-susceptibility in rice. In detail, they developed and analyzed an F2 population from the cross between a resistant *indica* variety and a susceptible *japonica* variety, through which they demonstrated co-segregation between the *Pid3* pseudogene and this susceptible phenotype.

In the case of genetic mapping studies, the number of NBS-LRR mapped genes is a limiting factor. Recently, Marone *et al.*[107] analyzed the nucleotide sequences of 2000 diversity arrays technology (DArT) markers that were developed in wheat and had previously been used for the construction of several genetic maps as anonymous markers. Very interestingly, they found that a high proportion of the DArT clones corresponded to or were located very near to sequences related to disease resistance in plants, and in particular to NBS-LRR genes and protein kinases, most of which contained an LRR domain. These data allowed them to position a high number of NBS-LRR genes on the wheat genetic maps. As an example, 40 markers that corresponded to *R* genes were positioned on the wheat A and B genomes in a consensus map developed recently [108]. In particular, the DArT marker that corresponds to an NBS-LRR gene (*wPt-1601*) represents the peak marker of a major QTL for resistance to soil-borne cereal mosaic virus that was mapped on chromosome 2B in the durum-wheat population Meridiano × Claudio [109]. Furthermore, Russo *et al.*[110] identified two markers that corresponded to NBS-LRR genes as peak markers of minor QTLs for the same disease on chromosomes 3B and 7B in another durum-wheat population (Cirillo × Neodur). Further evidence is available in the literature relating to the involvement of some DArT markers that correspond to NBS-LRR genes in plant disease resistance. The marker *wPt-8460* on chromosome 2B was shown to be significantly associated with stem-rust resistance by Yu *et al.*[111] based on an association mapping analysis. The sequence of this marker is not available, but it was positioned at 3 cM from the marker *wPt-0189* in a different segregating population of durum wheat (Creso × Pedroso) [107], which corresponds to an NBS-LRR gene. Similar examples are also available for other species of agronomic interest. Ashfield *et al.*[112] reported that CC-NBS-LRR genes co-segregate with the *Rpg1-b* locus, which confers resistance to strains of *P. syringae* pv. *glycinea* in soybean. NBS-LRR sequences were shown to co-localize to 11 resistance loci in potato [113], as the *Rpi-blb2* locus on chromosome VI [114] and the *Gpa2/Rx1* locus on chromosome XII [54].

Experimental evidence has suggested that NBS-encoding resistance-gene analogs are also involved in both qualitative and quantitative resistance in cotton. Indeed, He *et al.*[30] showed that resistance-gene analogs mapped on linkage group A4 co-localized with a QTL that confers resistance to the cotton bacterial blight pathogen *Xanthomonas campestris* pv. *malvacearum*, while other resistance-gene analogs positioned on chromosome 20b co-mapped with a previously identified qualitative locus for resistance to bacterial blight.

These kinds of studies are of great importance, because they provide candidate genes for several resistance loci and they provide valuable sources of closely linked molecular markers that can be used in marker-assisted selection.

### 4.2. Breeding for More Resistant Crops

The development of genetic resistance to biotic stress to obtain resistant cultivars that can still produce high yields and maintain excellent quality traits is the most efficient, cost effective and environmentally friendly approach to prevent the losses caused by plant pathogens. Plant breeders have traditionally approached breeding for resistance in successive steps, which have included: (i) screening of germplasm collections to identify sources of resistance, and characterization of their phenotypes; (ii) studying of the mode of inheritance; (iii) introgression of the resistance traits in elite cultivars; and (iv) assessment of the performance of the new cultivars under pathogen challenge in the field [115,116]. The identification of genetic markers for marker-assisted selection (MAS) can greatly shorten the duration of a breeding program, increase the selection efficiency, and limit the phenotypic assessment, which is often laborious and time consuming. An alternative approach to MAS is the development of transgenic plants that express *R* genes or pathogenesis-related genes. Some transgenic crops that carry resistance to diseases have been developed based on information relating to the barley *Rpg1* (reaction to *Puccinia graminis 1*), rice *Pi9*, *S. bulbocastanum RB2*, and soybean *Rps1-k* genes. All of these genes code for NBS-LRR proteins [117].

Two general categories of disease resistance are mainly used in breeding for the improvement of disease resistance: complete or qualitative resistance, which is usually controlled by a single *R* gene, and incomplete or quantitative resistance, which is dependent on multiple genes, each of which have only a partial effect. *R* genes typically provide high levels of resistance and are relatively easy to manipulate, both in basic research and in applied breeding programs. However, their use is often limited to a specific race of a pathogen. Furthermore, they are characterized by a lack of durability, due to the continuous evolution of the pathogens [118]. Instead, quantitative partial resistance tends to be more durable than typical *R*-gene-mediated resistance, due to the action of multiple loci and the broader specificity [119]. At the same time, the limitations caused by the loss of durability of single-gene-based resistance can be overcome by the combination of resistance genes; *i.e.*, by the incorporation of multiple *R* genes into single cultivars to achieve greater durability. This process is referred to as “gene pyramiding” or “gene stacking”. The use of molecular markers is particularly suitable for gene pyramiding, and in particular when different genes with similar phenotypes have to undergo introgression into the same genotype. A great number of alleles with different specificities in pathogen recognition have been identified for some *R* genes. The transgenic approach has recently been proposed as a tool to obtain plants expressing more than two alleles of an *R* gene. Briefly, transgenic plants with different alleles integrated at distinct genomic loci are produced, and then these plants are crossed and progenies carrying all of the alleles are selected by using allele-specific molecular markers. Such an approach is being used for the *Pm3* gene (a CNL) for resistance to powdery mildew in wheat, for which 17 resistance-promoting alleles have been identified [120,121].

A huge number of loci for resistance to pathogens have been mapped in many species, and a number of these are generally used with closely linked molecular markers in breeding programs. As an example, in publicly financed wheat breeding programs in the USA, Australia and Canada, about 50 genes are used in MAS for resistance to the main wheat diseases, which include powdery mildew, rusts, cereal cyst nematode, and viruses, and similar numbers of resistance genes are available in barley [122]. Nevertheless, the knowledge of the gene sequences linked to the resistance is very important, as this allows the design of perfect molecular markers that are not subject to the risk of recombination between the marker and the *R* gene.

Functional markers designed on NBS-LRR genes have been used to transfer *R* loci to susceptible genotypes. Bacterial blight, which is caused by *Xanthomonas oryzae* pv. *Oryzae* (Xoo), and blast, which is caused by the fungus *Magnaporthe grisea*, are the most devastating diseases that constrain rice production and food security [123]. To date, over 70 blast-resistance genes have been identified that are distributed across all of the chromosomes except chromosome 3, and many *R* genes are clustered in particular on chromosomes 6, 11 and 12 [124]. Over the last decade, 13 complete blast *R* genes have been cloned, and except for *Pi-d2*, all of these *R* genes encode NBS-LRR proteins. Interestingly, clusters that comprise a number of NBS-LRR genes are present in blast-resistance loci. As an example, the *Pi2/9* locus on chromosome 6 contains a complex cluster of NBS-LRR genes that have different specificities. Among these, there are three cloned genes: *Pi9*, *Pi2* and *Pit-z*. The large number of closely related NBS-LRR genes that are clustered in this region might contribute to the generation of new *R* alleles through recombination and uneven crossing-over, which will confer broad-spectrum resistance to different sets of blast strains collected from different countries. Furthermore, different NBS-LRR genes that are often present in the cluster can concur to produce a resistant phenotype [125]. Therefore, functional markers designed on the NBS-LRR genes identified in these resistance clusters will be valuable tools for MAS programs for the improvement of disease resistance to *M. grisea*. A cluster of six NBS-LRR genes is located in the same rice genomic clone in which the *Pi40(t)* gene was positioned. Highly stringent primer sets that were designed for these genes were used to select backcross lines in a MAS program for the improvement of the resistance of rice varieties [126]. A functional marker designed on an NBS-LRR gene linked to the *Pi-1* gene for resistance to the same pathogen was used for gene pyramiding of this gene and *Piz-5* into the same susceptible genotype. Furthermore, to add resistance to bacterial blight, the *Xa21* gene (which is not an NBS-LRR gene) was also added, using genetic transformation [127].

Many efforts have been made internationally to incorporate modern selection technologies into breeding programs. An example of this is the WHEAT CAP project (http://maswheat.ucdavis.edu/), which is aimed at preparing MAS protocols to incorporate valuable genes for many traits of interest into the best wheat breeding lines. These lines are being used in the WHEAT CAP project to deploy the targeted genes into thousands of lines through the use of high-throughput forward-breeding strategies [128]. For instance, more than 160 leaf (*Lr*), stem (*Sr*) and stripe (*Yr*) rust resistance genes have been found and characterized in common hexaploid wheat, tetraploid durum wheat, and many diploid wild wheat species [116]. Among these, three genes for leaf-rust resistance that confer race-specific resistance have been isolated: *Lr1* and *Lr10*, which originated from common wheat, and *Lr21*, which originated from *Triticum tauschii*[129–131]. These genes condition hypersensitive responses to isolates of *Puccinia triticina*, and they encode CNL proteins.

## 5. Conclusions

Studies of the NBS-LRR gene family in plants represent an intriguing challenge and can provide knowledge on the genomic and molecular mechanisms that form the basis of gene regulation and protein function. Their evolution at the gene and genomic level can be defined through this ancient and numerous gene family. Finally, the involvement of NBS-LRR genes in resistance to a plethora of pathogens makes these genes an invaluable source for the improvement of disease resistance in plants through the use of perfect molecular markers or through genetic transformation. In particular, the new genomic technologies can provide the chance to speed up the identification of genetic determinants of resistance to diseases in plants. Approaches that are based on next generation sequencing can help in the characterization of the numerous NBS-LRR gene family members in the plant genome, and in the identification of the associations between specific allelic forms and resistant phenotypes. Then, high-throughput genotyping platforms, such as those based on single nucleotide polymorphism chips, or in a very near future, genotype-by-sequencing, will strongly facilitate the selection of plants with the best allelic combinations at multiple loci.

## Figures and Tables

**Table 1:** Nucleotide-binding site (NBS)-encoding *R* genes and pseudogenes identified in different plant genomes.

Plant species	Total number of NBS-LRR genes	TNL *	CNL *	Number of pseudogenes	References
*Arabidopsis thaliana*	149	94	55	10	[20]
	159	98	50	-	[21]
*Populus trichocarpa*	402	91	119	161	[22]
*Medicago truncatula*	333	156	177	49	[19]
*Vitis vinifera*	459	97	203	-	[23]
*Oryza sativa L.* spp. *indica*	653	-	-	184	[14]
*Oryza sativa L.* spp*. japonica*	553	-	-	150	
*Carica papaya*	54	7	6	-	[12]
*Cucumis sativus*	57	13	18	-	[13]
*Brassica rapa*	92	62	30	-	[17]
*Lotus japonicus*	158	32	28	62	[18]
*Arabidopsis lyrata*	185	123	38	-	[21]
*Glycine max (*soybean)	319	-	-	-	[24]
*Brachypodium distachyon*	126	0	113	-	[9]
*Solanum tuberosum*	438	77	361	-	[15]
	435	65	370	179	[16]

* TNL and CNL group all of the NBS-LRR genes with TIR and CC motifs, respectively.
